# Genetic diversity and antibiotic resistance of *Mycobacterium bovis* in bovines in the Delta area of Egypt

**DOI:** 10.3389/fcimb.2025.1600225

**Published:** 2025-09-12

**Authors:** Mohamed Sabry A. Elsayed, Zahraa H. Alqaim, Aysam M. Fayed, Samah Mahmoud Eldsouky, Mohamed Salah Basiouny, Azza M. Metwally, Ahmed Abdelbadee, Al Shaimaa Hasan, Amira Kamal ElDin Mohammed ElAlfy, Mai Afifi Nasr, Shimaa Mostafa Elnahas Wahdan, Rasha Abdelhamid Elsayed, Mai Magdy Anwer, Abeer Mahmoud El-Bahy, Ahmed Salah

**Affiliations:** ^1^ Department of Bacteriology, Mycology, and Immunology, Faculty of Veterinary Medicine, University of Sadat City, Sadat City, Menoufia, Egypt; ^2^ Department of Anesthesia Techniques, College of Health and Medical Technique, Al-Mustaqbal University, Babylon, Iraq; ^3^ Department of Molecular Biology, Genetic Engineering and Biotechnology Research Institute, University of Sadat City, Sadat City, Minufiya, Egypt; ^4^ Medical Laboratory Techniques Department, College of Health and Medical Technique, Al-Mustaqbal University, Babylon, Iraq; ^5^ Department of Otolaryngology and Head and Neck Surgery, Benha University, Faculty of Medicine, Benha, Egypt; ^6^ School of Biotechnology, Badr University in Cairo (BUC), Badr, Cairo, Egypt; ^7^ Environmental Studies and Research Institute, Sadat City University, Sadat City, Egypt; ^8^ Department of Molecular Biology, Genetic Engineering and Biotechnology Research Institute, University of Sadat City, Sadat City, Menoufia, Egypt; ^9^ Animal Biotechnology Department, Genetic Engineering and Biotechnology Research Institute, University of Sadat City, Sadat City, Minufiya, Egypt; ^10^ Department of Medical Pharmacology, Qena Faculty of Medicine, South Valley University, Qena, Egypt; ^11^ Department of Internal Medicine, Faculty of Medicine, Benha University, Benha, Egypt; ^12^ Department of Chest Diseases, Benha University, Benha, Egypt; ^13^ Department of Medical Microbiology and Immunology, Benha University, Benha, Egypt; ^14^ Department of Public Health, Community, Environmental and Occupational Medicine, Benha University, Benha, Egypt; ^15^ Department of Hepatology, Gastroenterology and Infectious Diseases, Benha University, Faculty of Medicine, Benha, Egypt

**Keywords:** *Mycobacterium bovis*, cattle, buffaloes, antibiotic resistance, MIRU_VNTR genotyping

## Abstract

**Introduction:**

*Mycobacterium bovis* (*M. bovis*) causes significant financial harm to the cattle industry through decreased productivity and trade limitations, while also posing a risk to human health through zoonotic transmission, which is primarily from unpasteurized milk or close animal contact.

**Methods:**

Single intradermal tuberculin was used to test 2400 cases (1000 Holstein Friesian cattle and 1400 native breed buffaloes) during the national control program from Cairo, El-Buhaira, Dakahlia, Gharbia, Menoufia, and Sharkia districts located at the northern areas of Egypt. Tuberculin-positive cases were slaughtered and subjected to postmortem examination and isolation of *M. bovis* was performed. IS*6110* primer was used in PCR test to confirm the existence of genus mycobacterium and regions of difference-based differentiation was used to detect *M. bovis* on the species level, phenotypic and genotypic antimicrobial resistance, as well as mycobacterial interspersed repetitive unit-variable number tandem repeat analysis (MIRU-VNTR) were performed.

**Results:**

A total of 65 out of 2400 (2.7%) cases were single intradermal tuberculin test positive, 40 out of 65 (61.53%) were *M. bovis* positive on PCR, and the 40 isolates exhibited susceptibility to ethambutol, rifampicin, streptomycin, ciprofloxacin, levofloxacin, ofloxacin, and sparfloxacin. From them, 32 (80%) were susceptible to isoniazid, and 8 (20%) were resistant. These eight isolates contained three distinct *kat*G mutations at codons 315, 463, and 506 with rates of 2/8 (25%), 3/8 (37.5%), and 3/8 (37.5%), respectively each representing a unique, single-codon mutation. MIRU-VNTR analysis enabled the identification of 35 distinct genotypes, with genotypes 26, 27, and 28 showing high prevalence. The nine highly discriminatory loci MIRU10, QUB11b, MIRU26, QUB26, QUB4156, MIRU04 ETRD, ETRA, Mtub30, and Mtub39 with a discriminating index of 0.9676 were suitable for the preliminary genotyping of *M. bovis* isolates from animals. *M. bovis*, ID: 7540/01, Lineage: Bovis and ID: 951/01, Lineage: Bovis from Germany were the closest lineages to our genotypes using the MIRU-VNTR plus database.

**Conclusion:**

*M. bovis* isolated from cattle and buffaloes of some Delta area districts expressed high diversity and some isolates showed resistance to isoniazid with *kat*G mutations. Continuous implementation of MIRU-VNTR analysis will help to trace the origin and similarities among animal and human isolates within the Delta area.

## 1 Introduction

*Mycobacterium bovis* is considered the dominant cause of bovine tuberculosis (bTB), a progressive disease which affects cattle worldwide. Bovine tuberculosis was classified as a transmissible disease on list B by the World Organization for Animal Health (WOAH); and has significant implications on public health, food security and safety along with international trade (Langer and LoBue, 2014; Abdelaal et al., 2019).

The *M. tuberculosis* complex (MTBC) includes *M. bovis, M. tuberculosis, M. microti*, *M. caprae*, *M. mungi*, *M. pinnipedii*, *M. suricattae*, and *M. orygis* (Lombard et al., 2021). Species demarcation is the process of figuring out which individual organisms belong to the same species and which are distinct species. This complex issue has been a central concern in taxonomy from its very start, leading to the development of established procedures. Genomic data is rich with information about how genetically similar species are, including evidence of past and recent introgression. This makes it very useful for demarcating species based on various definitions.

Genetically, the MTBC species have similarities and relationships that have been investigated based on digital DNA-DNA hybridization (dDDH), next-generation sequencing (NGS), and average nucleotide identity (ANI). The scientific community generally accepts that mycobacterial species share enough genetic similarity to be grouped together if their dDDH reaches at least 80% and their ANI is at least 96%. The pairwise results showed that all of the MTBC species are exceedingly closely matched to each other (dDDH: 91.2-99.2%, ANI: 99.21-99.92%), well beyond the respective species demarcation thresholds, indicating that they are members of the same species.

It was suggested that *M. tuberculosis* is currently considered a single species, with other MTBC members being variants (Riojas et al., 2018). Moreover, these members have the same 16SrRNA sequence and up to 99.9% nucleotide similarity (Borham et al., 2022a).

In developing countries, human tuberculosis from *M. bovis* is common due to animal contact. Clinically, *M. bovis* and *M. tuberculosis* infections are indistinguishable (Torres-Gonzalez et al., 2016). Both *M. tuberculosis* and *M. bovis* can cause pulmonary, extrapulmonary, and disseminated diseases as well as primary and reactivated infections. Microorganisms can be transmitted in various ways, particularly through consumption of contaminated dairy and meat products from infected animals. *M. bovis* is commonly spread to the lymph nodes, joints, pleural space, eyes, abdominal organs such as liver, and central nervous system (Majoor et al., 2011; Sotoudeh et al., 2021). *M. tuberculosis* can cause several otorhinolaryngeal issues, including laryngeal tuberculosis, with typical hoarseness, odynophagia, and dysphagia, as well as weight loss and appetite loss. In severe cases, *M. tuberculosis* can lead to middle ear tuberculosis, which manifests as hearing loss, otalgia, otorrhea, and facial palsy. Moreover, nasal obstruction and bloody rhinorrhea are symptoms of paranasal and nasal tuberculosis (Michael and Michael, 2011). In addition, a 59-year-old woman with a 2-year-old scaly and erythematous ear lesion was diagnosed with skin TB due to infection by *M. bovis (* Lhote et al., 2016).

Infection with *M. bovis* in cattle is considered a chronic and debilitating disease causing granulomas in the head, thoracic lymph nodes, lungs, pleura, intestinal tract, liver, spleen and peritoneum being most commonly affected, however it can progress to affect any body tissue/organ (WOAH, 2024).

Because natural pastures are scarce in Egypt, the bovine industry takes place in closed farms and depends on food supplies coming from cultivated lands. Male animals and infertile females are fattened for meat, while female cattle and buffaloes are employed for milk production. There are three main cattle and buffalo production systems in Egypt: intensive, semi-intensive, and extensive. The intensive system accounts for 7% of the total cattle and buffalo population, with farms containing 10 to 1000 heads, the semi-intensive accounts for 60% of the total cattle and buffaloes with herds containing 1 to 50 cattle and buffalo heads, and the extensive accounts for 33% of the total cattle and buffaloes, with farmers keeping 1 to 10 heads. In Egypt, native cattle are known as “Baladi,” which means “local” with no genetic subdivisions. They are raised all across the country, are acclimatized to Egyptian circumstances, and have a great tolerance for endemic diseases. Due to these animals’ low milk productivity, genetic improvement programs have included crossbreeding with exotic high-producing cattle breeds such as Holstein, Brown Swiss, and Simmental, resulting in a large scattered population raised in small- or medium-sized herds by local farmers under the breed name “Mixed,” as described and characterized by the Ministry of Agriculture and Land Reclamation. Egyptian livestock imports have grown over the last decade, helping the dairy industry to operate better. The three primary countries from which Egypt imports live dairy cattle are Germany, the Netherlands, and the United States of America.

The Egyptian water buffalo species were found in the Nile Delta and Nile Valley and were known as Baladi or by other local names such as Beheri, Minufi, and Saidi. Egyptian buffalo are classified as either Riverine (black with long curled horns) or Swamp (dark grey, black, black and white, or pure white with long softly curved horns) (Ibrahim, 2012; Abou El-Amaiem, 2014; Hamada et al., 2020; Elsayed et al., 2022). According to the official Egyptian GOV bTB database from 2009 to 2013, the rate of infection among Egyptian cattle declined from 0.23 to 0.02 between 2009 and 2012, but increased to 0.082 in 2013 (Abdellrazeq et al., 2016). Positive skin test cases represented 3% of the animals in 40% of studied herds between 2013 and 2015 (Hamed et al., 2021). Between 2015 and 2018, the prevalence of bTB in 16 dairy farms in the districts of Upper Egypt, Alexandria Road, and mid-delta, was 1.67% (Elsohaby et al., 2020).

In 2018, there was a notable prevalence of *M. bovis* infection in cattle within the Nile Delta region of Egypt. One of the studies indicated a significant rate of infection, particularly in dairy herds, with herd-level prevalence reaching up to 41% and animal-level prevalence around 3.4% with single intradermal comparative skin tuberculin (Abdelaal et al., 2019). In two separate studies made in the Delta area of Egypt, Elsayed and Amer (2019) identified a 3.5% tuberculin-positive rate among buffalo and cattle (74 out of 2100 animals), with *M. bovis* accounting for 82.4% (61 out of 74) of these cases and *M. tuberculosis* for 2.7% (2 out of 74). In addition, mixed infection patterns involving both *M. bovis* and *M. tuberculosis* were observed in 8 out of 74 samples (10.8%) and the remaining 3 out of 74 samples (4.05%) were negative. A later study by Elsayed et al. (2022) reported a lower overall positive rate of 1.5% (54 out of 3700 animals), specifically 1.2% of buffalo and 1.6% of cattle, and confirmed *M. bovis* in 46.3% (25 out of 54) of the positive animals. Egypt’s rising demand for meat and milk, reaching 28.51kg per capita for meat and 53.1kg for milk in 2017 (FAO, 2017), contributes to *M. bovis* transmission. This is largely due to consumer habits like drinking unpasteurized (raw) milk and consuming dairy products made from it, such as cheese, yogurt, cream, and ice cream (Collins et al., 2022). Additionally, *M. bovis* can spread through meat due to practices like unofficial slaughtering outside slaughterhouses (especially during Eid al-Adha) and the common consumption of undercooked meat found in fast and takeaway foods. These habits have led to confirmed human cases of zoonotic tuberculosis caused by *M. bovis*, with antibiotic resistance verified through both phenotypic testing and whole-genome sequencing (Soliman et al., 2024).

Modern molecular epidemiological techniques have been widely applied to *M. tuberculosis*, revealing crucial information about the pathogen’s diversity, the disease’s origins, transmission dynamics, the co-evolution of the bacterium and humans, and the development of drug resistance (Jagielski et al., 2014; Niemann and Supply, 2014). By using the same molecular epidemiology methods developed for *M. tuberculosis*, we can gain valuable insights into the evolution and transmission dynamics of *M. bovis* in Egypt and the African continent. The results can be easily compared among various laboratories because they are presented in numbers and MIRU-VNTR typing has maximum efficiency and reproducibility. MIRU-VNTR typing, which depends on a collection of 24 loci, is used routinely in MTBC genotyping and is considered a global standard (Supply et al., 2006; Coll et al., 2014; Carvalho et al., 2016). *M. tuberculosis* genotyping approaches including MIRU-VNTR genotyping, spoligotyping, single-nucleotide polymorphisms, and whole-genome sequencing, were used to generate valid genotypic data for the *M. bovis* positive infections identified (Teeter et al., 2016; Franco et al., 2017).

Drug-resistant bacterial strains circulating in cattle, particularly those resistant to human tuberculosis medications, significantly raise the risk of widespread multidrug-resistant infections in people (Vázquez-Chacón et al., 2021). The global rise of multidrug resistance (MDR) in bacteria is a significant public health threat. Recent studies show an increase in MDR bacterial pathogens from various sources, highlighting the urgent need for responsible antibiotic use. It’s also crucial to routinely perform antimicrobial susceptibility testing to identify effective antibiotics and to screen for emerging MDR strains (Dos Anjos et al., 2022).

In Egypt, there’s minimal concern about antimycobacterial drug residues in meat from *M. bovis*-infected animals meant for human consumption. This is because infected animals are typically tested and slaughtered rather than treated and should be not passed for human consumption until subjected to thorough postmortem inspection. However, if, for some reason, an animal were to be treated for *M. bovis* (e.g., in a non-food producing animal or in a research setting, or in countries with different control policies), strict adherence to established withdrawal periods for the specific drugs used would be paramount before any part of the animal could enter the food chain.

To the best of our knowledge, isoniazid (INH) and rifampin (RIF) resistant *M. bovis* isolates from cattle have been found in Brazil and Italy (Djemal et al., 2018). In 2019, a study in the Nile Delta found that 18.2% (2 out of 11) of *M. bovis* isolates were resistant to Isoniazid, while the remaining isolates were sensitive to all tested drugs such as isoniazid, rifampicin, ethambutol, and streptomycin (Abdelaal et al., 2019). Abdelsadek et al. (2020) reported that 59.3% of multidrug-resistant *M. tuberculosis* and *M. bovis* strains, sampled from Egyptian Delta farm animals, veterinarians, and workers, showed isoniazid resistance. El-Gedawy et al. (2024) reported that 30.8% of *M. bovis* isolated from cattle in specific Delta districts were isoniazid-resistant. Mycobacterial *kat*G protects against reactive oxygen, helps survival in macrophages, and is a key to isoniazid’s mechanism. Thus, isoniazid resistance often stems from *kat*G loss of function due to gene deletion or point mutations, as seen in most resistant *M. tuberculosis* strains (Kang et al., 2006). Soliman et al. (2024) proposed that a point mutation in codon 463 of the *kat*G gene is a primary cause of isoniazid resistance in *M. bovis* isolates from zoonotic tuberculosis in Egypt. Thus, monitoring antimicrobial resistance in *M. bovis* from animals may help in human treatment if zoonotic transmission of *M. bovis* from animals to human beings took place.

This study aimed to investigate the genetic diversity of *M. bovis* isolates from cattle and water buffalo in the districts of the Delta region in Egypt using MIRU-VNTR analysis. Furthermore, the study aimed to detect the similarity between the local and international genotypes, assess the susceptibility of *M. bovis* isolates to eight antitubercular drugs, and elucidate the genotypic basis of resistance to the four following commonly used antimicrobials: rifampicin, isoniazid, streptomycin, and ethambutol through detection of accumulated genetic mutations. Finally, other goals of this study included: 1) supporting government authorities with access to the tools necessary for rapid and accurate diagnosis of *M. bovis* infections, 2) provide a list of effective antimicrobials if *M. bovis* zoonotic transmission occurred in humans within these governorates of Egypt as well as 3) potential treatment strategies for successful control and prevention of disease spread.

## 2 Methods

### 2.1 Tuberculin testing

**Figure 1:**
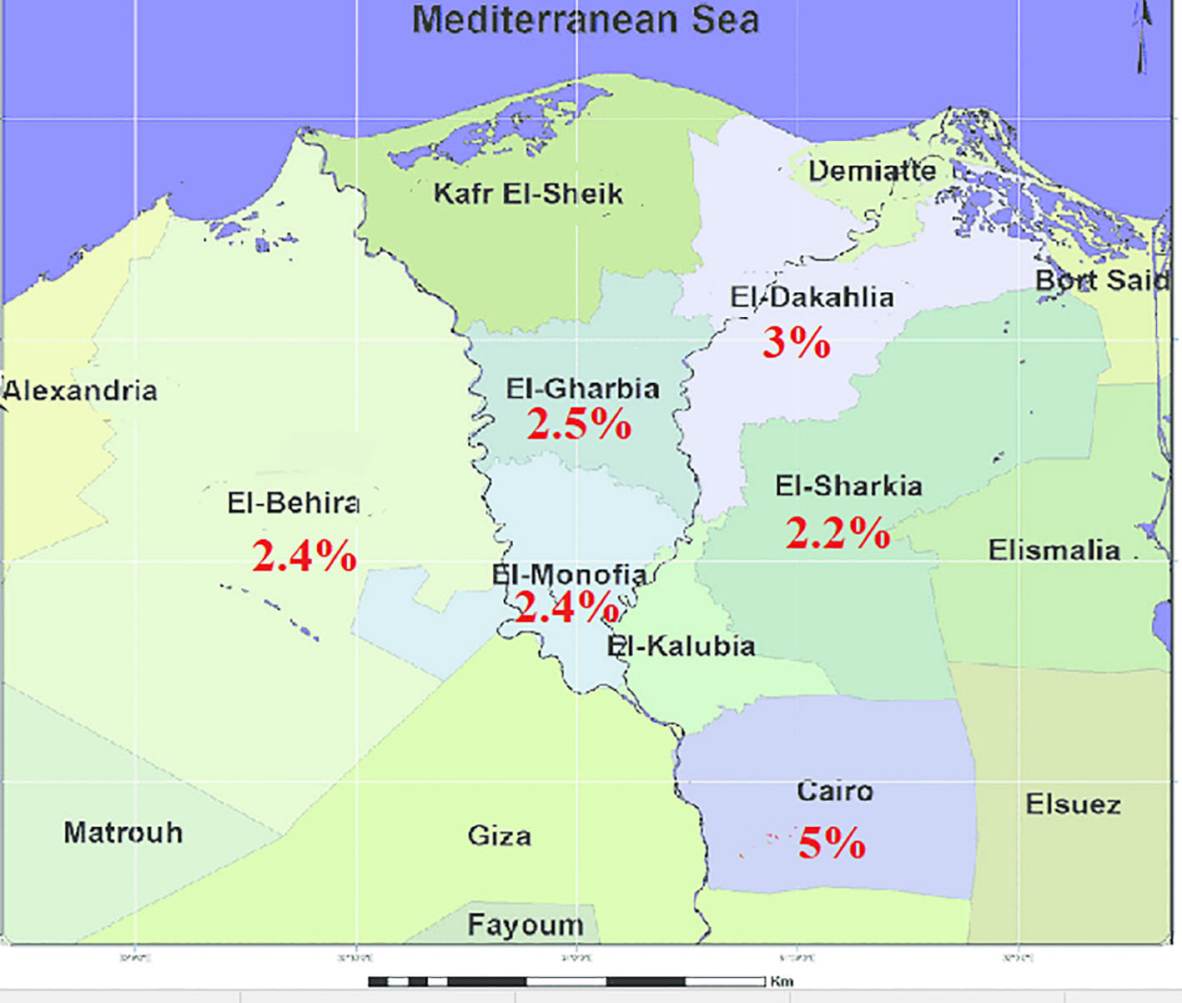
Map showing sites and distances among the tested Egyptian districts and percentages are for tuberculin-positive cases per district.

A total of 2400 animals (1400 Holstein Friesian cattle and 1000 native breed buffaloes) were tested using a single intradermal tuberculin test between 2019 and 2021. The tested cattle and buffaloes for each surveyed Egyptian district were distributed as follows: in Cairo were (100 and 100), El-Buhaira were (350 and 150), Dakahlia were (300 and 100), Gharbia were (200 and 200), Menoufia were (250 and 200), and Sharkia were (200 and 250). These animals were tested during the national screening program. Buffalo and cattle that were owned by the same farmers and kept together in the same groups were simultaneously incorporated in this study. The site and distances among the screened Egyptian districts are shown in **Figure 1**. A single intradermal tuberculin test was performed via the intradermal injection of bovine purified protein derivative (PPD), and testing and interpretation were performed according to the WOAH guidelines (WOAH, 2024). Although some of the tested cases had been previously screened with a single intradermal tuberculin test and given a negative result one year earlier, the majority were new cases due to animal purchasing and movement. The national control program covers all Egyptian rearing system types which consist of intensive, semi-intensive, and extensive. Due to the lack of sufficient funding, there was no ability to cover all of Egypt in this study. The animals were tested during the national control program in these Delta area districts which targeted animals owned by farmers (rearing small numbers) in the villages. Due to the variations of animal numbers owned by these farmers, there were no specific herd names and numbers. Each tested case was documented with data points including animal characteristics (such as its number, species, sex, age, and pregnancy state) and owner identifiers (name, identification card number, address, and mobile contact). It was particularly important to focus on testing this sector as these herds may have an insufficient biosecurity plan, more lax hygiene measures, increased possibility of direct contact between farmers and animals, and a decreased implementation of preventive measures for animal excreta and products. In Egypt it is easier to take quarantine measures on small and large herds rather than on the animals reared by farmers in the villages. The recent self-declaration of the chairman of the general organization of Veterinary Services of Egypt indicated that the large cattle farms from Egypt are considered free from MTBC infections from 2019 to 2021 (WOAH, 2022). Therefore applying a test and slaughter policy may help in decreasing the rate and spread of bTB infection in animals reared by farmers in the villages of the Delta area districts. This would require a good identification system of individual animals.

### 2.2 Sampling and cultivation

#### 2.2.1 Collection of samples

According to Egyptian law, all animals that test positive on single intradermal tuberculin test are sent to slaughter followed by postmortem examination. The mandibular, retropharyngeal (lateral and medial), left and right bronchial, cranial and caudal mediastinal, hepatic and mesenteric lymph nodes as well as the tonsils were examined and collected. Samples were also taken from the intestines, liver, lungs, and spleen. Based on whether a lesion was apparent after dissecting and visually checking target tissues, animals were assigned to either the visible-lesion or non-visible-lesion category. The samples from every case were combined into a single collective sample during the grinding process and then decontaminated and concentrated using Petroff’s method to cultivate and isolate *Mycobacterium* species (Mackie and McCartney, 1989). The study design and all the experimental protocols were approved by the Institutional Committee for Animal Care and Use, at the Faculty of Veterinary Medicine, University of Sadat City, Egypt, and the approval number was 2019-30. Herein we confirm that all methods were carried out following the relevant guidelines and regulations. All the methods are reported in accordance with ARRIVE guidelines (https://arriveguidelines.org) for the reporting of animal experiments.

#### 2.2.2 *M. bovis* isolation and identification

Every sample was grown in four tubes of Lowenstein-Jensen (LJ) media (bought from Thermo Fisher Scientific, Waltham, MA, USA): two of them with sodium pyruvate and two tubes with glycerol (acquired from Thermo Fisher Scientific, Waltham, MA, USA) (ABID AG, Austria). Two tubes one containing pyruvate and one with glycerol supplements were subjected to incubation in light, while the remaining tubes were incubated in dark conditions at 37 °C with 5% CO_2_. We monitored the bacterial growth rate by watching the cultivated tubes daily and used Ziehl-Neelsen staining to identify acid-fast bacilli (Romano et al., 2005; Elsayed, 2019).

### 2.3 DNA extraction and molecular confirmation of isolates

The DNA extraction was implemented using the QIAamp DNA extraction Miniprep Kit (Qiagen, Germany) based on the manufacturer’s recommendations for Gram-positive bacteria. The M. bovis isolates were killed using a 2:1 combination of chloroform and methanol purchased from CLN GmbH (Germany). A few colonies were placed in 1.5 mL Eppendorf tubes, 200 µL Tris EDTA (TE) was added, and the tubes were frozen overnight. After freezing, 200 µL of freshly prepared chloroform: methanol mixture was added. The tubes were centrifuged at 2,000 × g for 20 min before decanting the supernatant. The tubes were then centrifuged at 6,000 × g for 5 min before discarding any remaining supernatant. Finally, the tubes were air dried for 1 h before being frozen until DNA extraction could begin. DNA was extracted using a combination of chemical, physical, and enzymatic techniques (Warren et al., 2006).

#### 2.3.1 *IS*6110 confirmation and RD-based differentiation of *M. bovis*

##### 2.3.1.1 *IS*6110 is an insertion sequence that is a repetitive mobile element which is unique to the *Mycobacterium tuberculosis* complex

Regions of difference (RDs) in mycobacterial genomes are classified as either phylogenetically informative or non-informative. The latter, including Proline-Glutamic acid (PE)/Proline-Proline-Glutamic acid (PPE) protein genes, prophages, and insertion sequence-flanked regions, are highly variable and strain-specific due to recombination (Ru et al., 2017). Specific RDs (like RD1, RD4, RD9, RD12) are used to differentiate members of the *Mycobacterium tuberculosis* complex based on their presence or absence and PCR product sizes (Warren et al., 2006). The 9.5-kb RD1 segment is crucial, present in virulent *M. tuberculosis* but absent in attenuated *M. bovis* BCG strains. It encodes at least nine proteins, including ESAT-6 and CFP-10, which are significant as potential virulence factors, vaccine candidates, and diagnostic markers due to their strong antigenic properties to T and B cells (Daugelat et al., 2003). ESAT-6 directly increases membrane permeability, suggesting a role in cellular damage. Both ESAT-6 and CFP-10 influence TNF-α production in macrophages, a cytokine that can induce cell death in infected macrophages. Restoring the RD1 region in BCG enhances its virulence and immunogenicity by re-expressing CFP10 and ESAT6 (Guo et al., 2012).

All primers used in this study (**Table 1**) were obtained from Takara, Japan. PCR methodology and conditions were used for a total PCR volume of 25 µL: 1 µL DNA sample (typically 10 ng, and the final concentration was 200 pg/µL), 2 µL of 25-mM MgCl2 (Sigma-Aldrich, UK), 4 µL of 10-mM dNTPs (Sigma-Aldrich, UK), 5 µL of high GC buffer (Takara Holdings, Japan), 0.5 µL each primer (50-pmol/L), 0.125 µL HotStar Taq DNA polymerase (Sigma-Aldrich, UK), and the volume was completed with RNAse-free H_2_O (Sigma-Aldrich, UK). Amplification started with a 5 min incubation at 95 °C, followed by 45 cycles of 94 °C for 1 min; the annealing temperature was 65 °C for 1 min for IS6110 and 62 °C for 1 min for RD-based differentiation, and72 °C for 1 min. PCR tubes were maintained at 72 °C for 10 min (Palomino et al., 2002; Thacker et al., 2011). The PCR conditions were standardized according to the listed references. The positive control was *M. bovis* (ATCC35734D-2) and the negative control was *E. coli* (ATCC 11775).

**Table 1:** The primers of the targeted genes in this study.

Target gene	Primer sequences (5′-3′)	Amplicon size (bp)	Annealing temperature (°C)	Accession number	Reference
IS*6110*	5’-AGTTTGGTCATCAGCCGTTC-3’ 5’-CGAACTCAAGGAGCACATCA-3’	334	65	NC000962.3	Thacker et al. (2011)
RD1	5′-AAGCGGTTGCCGCCGACCGACC-3′ 5′-CTGGCTATATTCCTGGGCCCGG-3′ 5′-GAGGCGATCTGGCGGTTTGGGG-3′	146	62	CP009186.1	Warren et al. (2006)
RD4	5′-ATGTGCGAGCTGAGCGATG-3′ 5′-TGTACTATGCTGACCCATGCG-3′ 5′-AAAGGAGCACCATCGTCCAC-3′	172		CP009186.1	
RD9	5′-CAAGTTGCCGTTTCGAGCC-3′ 5′-CAATGTTTGTTGCGCTGC-3′ 5′-GCTACCCTCGACCAAGTGTT-3′	235		CP009186.1	
RD12	5′-GGGAGCCCAGCATTTACCTC-3′ 5′-GTGTTGCGGGAATTACTCGG-3′ 5′-AGCAGGAGCGGTTGGATATTC-3′	369		CP009186.1	

### 2.4 Drug susceptibility of *M. bovis* isolates using broth dilution methods

The resazurin microtiter assay plate was used to test the antibiotic susceptibility of the 40 *M. bovis* isolates. As previously described, the test was conducted in triplicate in a 96-well plate (Nunc Edge 2.0 96-well plate, Thermo Fisher Scientific, USA). In brief, the drugs used were isoniazid (INH), ethambutol (EMB), rifampicin (RIF), streptomycin (STR), ciprofloxacin (CPFX), levofloxacin (LVX), ofloxacin (OFX), and sparfloxacin (SPFX) purchased from Sigma-Aldrich, UK. The drug concentration was 1 mg/mL in distilled water, sterilized via filtration, and frozen until use. The antibiotic solutions were thawed and diluted in Middlebrook 7H9 medium supplemented with oleic acid albumin dextrose catalase (OADC). Serial twofold dilutions of each drug in 100 μLof medium to reach a final concentration of 0.5-5 μg/mL. Each well was inoculated with 100 μL of Middlebrook 7H9 medium supplemented with OADC with the diluted antibiotics as well as 100 μL of bacterial suspension that was adjusted to a 1.0 McFarland tube and diluted 1:20 in the same medium. The plates were sealed with lids, placed in a plastic bag, and incubated at 37 °C for 7 days. Then, 30 µl of freshly prepared 0.01% resazurin solution (Acros Organics, USA) was added to each well. Color development was observed after the plates were incubated overnight at 37 °C. For isolates that indicated either resistance or an ambiguous chromatic modification, the test was done twice. Drug-sensitive *M. bovis* isolates served as negative internal controls, whereas drug-resistant *M. bovis* isolates served as positive internal controls (Cingolani et al., 1999).

### 2.5 Detection of mutations

The isoniazid resistance-specific region of *kat*G was sequenced to identify mutations. The previously published primers by Cingolani et al. (1999) with their sequence listed in **Supplementary Table 3** was synthesized by Takara Holdings (Japan); the annealing temperature was 55 °C for 35 cycles, and the expected amplicon size was 337 bp. Although eight isolates were isoniazid-resistant, specific gene sequencing was performed to confirm that the phenotypic and genotypic characteristics matched. In brief, the PCR mixture contained the following reagents in a total volume of 50 µL: 10 mM Tris-HCl (pH 8.0), 50 mM KCl, 1.5 mM MgCl2, 10% glycerol, 200 mM dNTPs, 1.25 U Taq polymerase (Perkin Elmer, Norwalk, CT), and 2.5-µL aliquots of template DNA. Purification of the amplified products was performed by the QIAquick PCR purification kit (Qiagen, Courtaboeuf, France) and sequenced using the Big Dye Terminator Cycle Sequencing Kit (Applied Biosystems Inc., Japan) on an ABI Prism 3100 genetic analyzer (Applied BiosystemsInc.). To investigate the presence or absence of mutations, all generated sequences were compared with the published sequences of the drug-sensitive *M. bovis* AF2122/97 reference strain. The free online software Clustal Omega, available at https://www.ebi.ac.uk/Tools/msa/clustalo/, was used for multiple sequence alignment and mutation detection. The DNA sequences in this study were deposited into the sequence read archive database with accession numbers BioProject: PRJNA795887, SRX13791085, SRX13791086, and SRX13791087.

### 2.6 MIRU–VNTR typing, allelic diversity (h), and discriminating index

#### 2.6.1 PCR protocol and primers utilized for MIRU–VNTR typing

The primers implemented for MIRU-VNTR typing of *M. bovis* and *M. tuberculosis* H37Rv were notably similar (Kim et al., 2010; Carvalho et al., 2016). The first eight primers (**Table 2**) used in this study targeted the following loci: 424, 960, 1955, 2163b, 2996, 3192, 4052, and 4156 with correspondent alias; Mtub04, MIRU10, Mtub21, QUB11b, MIRU26, MIRU31, QUB 26, and QUB4156. Furthermore, the remaining seven primers targeted the following loci; 580, 1644, 802, 2156, 577, 2401, and 3690 with correspondent aliases; MIRU 04, MIRU 16, MIRU 40, ETRA, ETRC, Mtub30, and Mtub39; these primers were obtained from Takara Holdings (Japan). The primer sequences are listed in **Table 2**.

**Table 2:** The primers of the MIRU-VNTR analysis applied within this study.

Target gene	Primer sequence	Amplicon size (unit bp)	Annealing temperature	Accession number
424: Mtub04	5’-CTTGGCCGGCATCAAGCGCATTATT-3 5’-GGCAGCAGAGCCCGGGATTCTTC-3′	51	66 °C	NC_000962.3
960: MIRU10	5’-GTTCTTGACCAACTGCAGTCGTCC-3′ 5’-GCCACCTTGGTGATCAGCTACCT-3′	53	65 °C	NC_000962.3
1955: Mtub21	5’-AGACGTCAGATCCCA GTT-3′ 5’-ACCCGACAACAAGCCCA-3′	57	63 °C	NC_000962.3
2163b:QUB11b	5’-CCGATGTAGCCCGTGAAGA-3′ 5’-AGGGTCTGATTGGCTACTCA -3′	69	63 °C	NC_000962.3
2996: MIRU26	5’-TAGGTCTACCGTCGAAATCTGTGAC-3′ 5’-CATAGGCGACCAGGCGAATAG-3′	51	63 °C	NC_000962.3
3192:MIRU31	5’-ACTGATTGGCTTCATACGGCTTTA-3′ 5’- GTGCCGACGTGGTCTTGAT-3′	53	63 °C	NC_000962.3
4052:QUB26	5’-GTGCCGGCCAGGTCCTTC C-3′ 5’- CACCGCGTGTTTGACCCGAAC-3′	111	65 °C	NC_000962.3
4156:QUB4156	5’-ACCGCAAGGCTGATGATCC-3′ 5’- GTGCATCTCGTCGACTTCC-3′	59	63 °C	NC_000962.3
580: MIRU04	5′-GCG CGAGAGCCCGAACTGC-3′ 5′-GCGCAGCAGAAACGTCAGC-3′	77	66 °C	NC_000962.3
1644: MIRU16	5′-TCGGTGATCGGGTCCAGTCCAAGTA-3′ 5′-CCCGTCGTGCAGCCCTGGTAC-3′	53	67 °C	NC_000962.3
802: MIRU40	5′-GGGTTGCTGGATGACAACGTGT-3′ 5′-GGGTGATCTCGGCGAAATCAGATA-3′	54	63 °C	NC_000962.3
2156:ETRA	5′-AAATCGGTCCCATCACCTTCTTAT-3′ 5′-CGAAGCCTGGGGTGCCCGCGATTT-3′	75	65 °C	NC_000962.3
577:ETRC	5′-GTGAGTCGCTGCAGAACCTGCAG-3′ 5′-GGCGTCTTGACCTCCACG AGTG-3′	58	65 °C	NC_000962.3
2401: Mtub30	5′-CGTCGTCGCCGAGCTGGATT-3′ 5′-CACCGGGGCTGGCAG CTAAG-3′	58	65 °C	NC_000962.3
3690: Mtub39	5′-CGGTGGAGGCGATGAACGTCTTC-3′ 5′-TAGAGCGGCACGGGGGAAAGCTTAG-3′	58	67 °C	NC_000962.3

The final reaction volume was 25 μl and consisted of 0.125 μl of HotStarTaq DNA polymerase (Sigma-Aldrich, UK), 0.5 μl of each primer (50 pmol/μl), 1 μl of template DNA, 2 μl of 25 mM MgCl2 (Sigma-Aldrich, UK), 5 μl of high GC buffer (Takara Holdings, Japan), 4 μl of 10 mM dNTPs (Sigma-Aldrich, UK), and the volume was completed using RNAse-free H_2_O (Sigma-Aldrich, UK). The PCR conditions included a hot start at 94 °C for 5 min, 35 cycles of denaturation at 94 °C for 30 s, annealing at 60 °C for 30 s, extension at 72 °C for 30 s, and a final extension at 72 °C for 7 min (Carvalho et al., 2016).

Free online software (https://blast.ncbi.nlm.nih.gov/Blast.cgi?PROGRAM=blastn&PAGETYPE=BlastSearch&LINKLOC=blasthome) was used to determine prospective target regions and accession numbers. Furthermore, the melting temperatures were detected using the online software OligoAnalyzer 3.1 (Integrated DNA Technologies, Inc.: https://eu.idtdna.com/calc/analyzer). The QIAxcel machine was used for automated electrophoresis and its software was used for data analysis. DNA extracted from *M. tuberculosis* H37RV was implemented as the control positive to calculate the numerical data of allele repeats for each locus. Furthermore, *E. coli* ATCC 25922 DNA was implemented as the control negative for the PCR reaction.

### 2.7 MIRU–VNTR typing, allelic diversity (h), and discriminating index calculations

The allele diversity (h) of various applied MIRU–VNTR loci was calculated using the equation h = n (1 − Σxi2)/(n 1), as n represents the total number of isolates and xi represents the frequency of the ith allele at the individual site and calculations were performed after the following formula to calculate the DI:


$$\text{DI}=1‐1/\text{N}\left(\text{N}‐1\right)\sum_{j=1}^{S}\text{Nj }\left(\text{Nj }‐1\right)$$

It establishes the likelihood that MIRU–VNTR typing will divide two unrelated isolates from a microbiological population into various groups (Hunter and Gaston, 1988; Müller et al., 2013). DIs and allele diversity for all tested VNTR loci and combinations were completed using web tools at https://www.miru-vntrplus.org/MIRU/index.faces. In addition, the discriminatory power for each used MIRU–VNTR locus was calculated online at http://insilico.ehu.es/mini_tools/discriminatory_power/index.php.

### 2.8 Data analysis

The significance of the differences between tuberculin test results (rates of positive cases from different districts), visible and non-visible lesion cases on the enhanced postmortem at time of slaughter, isolation results on Lowenstein–Jensen (LJ) medium with sodium pyruvate and with glycerol, and phenotypic antimicrobial susceptibility test results were calculated using the free online *post hoc* Tukey’s honestly significant difference (HSD) calculator available at https://astatsa.com/OneWay_Anova_with_TukeyHSD/. For calculating the clustering results for each locus combination and drawing the dendrograms, the online tools at the site https://www.miru-vntrplus.org/MIRU/index.faces were used. In addition, the discriminatory power calculator presented online at http://insilico.ehu.es/mini_tools/discriminatory_power/index.php was used to calculate the discriminatory power for each combination of MIRU–VNTR loci. Moreover, the 95% confidence interval for each DI result was calculated by the free online tools at http://www.comparingpartitions.info/?link=ToolI. Moreover, the 95% CI for each DI result was calculated by the free online tools at http://www.comparingpartitions.info/?link=ToolI.

## 3 Results

### 3.1 Results of tuberculin and isolation in relation to the geographical distribution of isolates

Of all tested cases, 65 out of 2400 (2.7%) were tuberculin-positive. 43 out of 65 (3.1%) cases were in cattle and 22 out of 65 (2.2%) cases were in buffalo. The positive cases were slaughtered at different slaughterhouses and examined for the existence of granulomas in lymph nodes and organ tissues as described previously in the methodology. The tuberculin test and postmortem examination results from different districts were distributed as follows: 10/200 (5%) cases from Cairo, 10/450 (2.2%) from Sharkia, 11/450 (2.4%) from Menoufia, 12/400 (3%) from Dakahlia, and 12/500 (2.4%) from El-Buhaira had generalized lesions at postmortem and 10/400 (2.5%) cases from Gharbia had pulmonary lesions at postmortem. A total of 40 out of 65 (61.53%) cases showed growth on Lowenstein–Jensen (LJ) medium with sodium pyruvate [cattle cases represented 23 out of 40 (57.5%) and buffalo cases were 17 out of 40 (42.5%)]. In contrast, no isolates showed growth on Lowenstein–Jensen (LJ) with glycerol. A total of 25 out of 65 (38.5%) cases showed no growth at all, 7 out of 40 (28%) were cattle and 18 out of 40 (72%) were buffalo, (**Table 3**). A site map showing the tested districts and their tuberculin-positive results are indicated in **Figure 1**. A significant difference was observed (p< 0.0001) between tuberculin test results from cattle and buffaloes, tuberculin test results and enhanced postmortem examination from screened districts, isolation rates on (LJ) medium with sodium pyruvate from cattle and buffaloes, and the negative isolation results on the same media between cattle and buffaloes.

**Table 3:** Results of tuberculin testing, post-mortem examination, and isolation.

Locality and no. of tested animals	Species		Tuberculin test results					Lesions	Isolation Lowenstein-Jensen (LJ)			Negative culture (25)	
	Cattle	Buffalo							With sodium pyruvate (40)		With glycerol		
			Positive		%		Total						
			Cattle	Buffalo	Cattle	Buffalo			Cattle	Buffalo		Cattle	Buffalo
Cairo (200)	100	100	6	4	6%	4%	10 (5%)	Generalized	3	2	0	1	4
Sharkia (450)	200	250	5	5	2.5%	2%	10 (2.2%)	Generalized	2	1	0	2	5
Menoufia (450)	250	200	7	4	2.8%	2%	11 (2.4%)	Generalized	5	4	0	0	2
Gharbia (400)	200	200	6	4	3%	2%	10 (2.5%)	Pulmonary	5	3	0	1	1
Dakahlia (400)	300	100	10	2	3.3%	2%	12 (3%)	Generalized	4	4	0	1	3
El-Buhaira (500)	350	150	9	3	2.5%	2%	12 (2.4%)	Generalized	4	3	0	2	3
Total	1400	1000	43 (3.1%)	22 (2.2%)			65 (2.7%)		23 (57.5%)	17 (42.5%)	0/65 (0.0%)	7 (28%)	18 (72%)

### 3.2 Molecular confirmation of the obtained isolates

All 40 isolates were confirmed as MTBC by testing 100% positive for the IS*6110* PCR primer. Of these, 36 (90%) came from cattle, with varying percentages from different districts: Cairo (5/5, 100%), Sharkia (2/3, 66.7%), Menoufia (7/9, 77.8%), Gharbia (8/8, 100%), Dakahlia (8/8,100%), and El-Buhaira (6/7, 85.7%). The remaining 4 isolates (10%) were from buffaloes, specifically from Sharkia (1/3, 33.3%), Menoufia (2/9, 22.2%), and El-Buhaira (1/7, 14.3%). Next, using a simplex polymerase chain reaction (PCR) to differentiate isolates using genomic regions of difference (RDs) (RD1, 4, 9, and 12), it was determined that all isolates were *M. bovis* (**Table 4**). Typically, *M. bovis* isolates displayed RD1 at 146 bp, but lacked RD4, RD9, and RD12 at their expected sizes of 268 bp, 108 bp, and 306 bp, respectively. Conventional PCR utilizing *IS*6110-targeting primers and RD-based detection of *M. bovis* confirmed the isolation results and proved that all 40 isolates (100%) were *M. bovis*.

**Table 4:** Molecular confirmation using IS*6110* and regions of difference.

Locality	Isolates (40)	Animal origin		IS*6110*	Regions of difference (RD) based primers for confirmation of *M. bovis*			
		Cattle	Buffalo		RD1	RD4	RD9	RD12
Cairo	5/40 (12.5%)	5/5 (100%)	0/5 (0.0%)	5/5 (100%)	present (146 bp)	RD4 absent (268 bp)	RD9 absent (108 bp)	RD12 absent (306 bp)
Sharkia	3/40 (7.5%)	2/3 (66.7%)	1/3 (33.3%)	3/3 (100%)				
Menoufia	9/40 (22.5%)	7/9 (77.8%)	2/9 (22.2%)	9/9 (100%)				
Gharbia	8/40 (20%)	8/8 (100%)	0/8 (0.0%)	8/8 (100%)				
Dakahlia	8/40 (20%)	8/8 (100%)	0/8 (0.0%)	8/8 (100%)				
El-Buhaira	7/40 (17.5%)	6/7 (85.7%)	1/7 (14.3%)	6/6 (100%)				
Total	40/65 (61.53%)	36/40 (90%)	4/40 (10%)	40/40 (100%)	40/40 (100%)			

### 3.3 Phenotypic detection of antibiotic resistance of the obtained isolates

All the isolates 40 out of 40 (100%) were sensitive to rifampicin, ethambutol, and streptomycin. Most isolates (32 out of 40, 80%) were susceptible to isoniazid, while the remaining eight out of 40 (20%) isolates were resistant (**Supplementary Table 1**). There was a significant difference (p< 0.0001) between the susceptibility results of isoniazid, rifampicin, ethambutol, and streptomycin. Furthermore, sensitivity to quinolones, such as ciprofloxacin (CPFX, 1 μg/mL), levofloxacin (LVX, 0.5 μg/mL), ofloxacin (OFX, 1 μg/mL), and sparfloxacin (SPFX, 1 μg/mL), was confirmed; all isolates 40 out of 40 (100%) expressed sensitivity to all of the quinolones (**Supplementary Table 2**). All tested quinolones demonstrated efficacy at 1 μg/mL, except levofloxacin, which demonstrated efficacy at 0.5 μg/mL.

### 3.4 Genetic detection of the isoniazid, rifampicin, ethambutol, and streptomycin resistance genes and sequencing of the PCR products

Sequencing of the PCR products enabled the detection of mutations. All eight phenotypically isoniazid-resistant isolates expressed three different *kat*G mutations; both cattle isolates from Cairo showed mutations in the codon 315 with an AGC→ACC change. In contrast, one cattle isolate from Sharkia and two cattle isolates from Menoufia exhibited a mutation in the codon 463 and a CTG→CGG change. In addition, cattle isolates from Gharbia, Dakahlia, and El-Buhaira exhibited the same mutation at codon 506 with a GAG→AAG change. After sequencing the *inh*A regulatory region, no mutations were found, and no mutations were detected in the *oxy*R-*ahp*C allele, which was similar to the wild type in all isolates. The sequencing results of *rpo*B for rifampicin, *emb* for ethambutol, and both *rps*L and *rrs* for streptomycin confirmed that these alleles were identical to their wild-type counterparts and that the eight isolates of *M. bovis* were sensitive to these antimicrobials (**Table 5**). These results confirmed genetic resistance to isoniazid only.

**Table 5:** Results of drug-resistant *M. bovis* isolates with mutations showing isoniazid resistance.

Locality	Animal species	Isolate code	Antimicrobial susceptibility testing										
			Isoniazid				Rifampicin		Ethambutol		Streptomycin		
			Phenotype	*kat*G mutation	*inh*A	*oxy*R-*ahp*C	Phenotype	*rpo*B mutation	Phenotype	*emb* mutation	Phenotype	*rps*L mutation	*Rrs* mutation
Cairo	Cattle	4	R	Codon 315 (AGC-ACC)	WT	WT	S	WT	S	WT	S	WT	WT
	Cattle	5	R	Codon 315 (AGC-ACC)	WT	WT	S	WT	S	WT	S	WT	WT
Sharkia	Cattle	8	R	Codon 463 (CTG-CGG)	WT	WT	S	WT	S	WT	S	WT	WT
Menoufia	Cattle	16	R	Codon 463 (CTG-CGG)	WT	WT	S	WT	S	WT	S	WT	WT
	Cattle	17	R	Codon 463 (CTG-CGG)	WT	WT	S	WT	S	WT	S	WT	WT
Gharbia	Cattle	25	R	Codon 506 (GAG-AAG)	WT	WT	S	WT	S	WT	S	WT	WT
Dakahlia	Cattle	29	R	Codon 506 (GAG-AAG)	WT	WT	S	WT	S	WT	S	WT	WT
El-Buhaira	Cattle	34	R	Codon 506 (GAG-AAG)	WT	WT	S	WT	S	WT	S	WT	WT

• R, Resistant.

• S, Sensitive.

WT, Wild type.

### 3.5 MIRU–VNTR loci analysis

As shown in **Table 6**, the loci 4156:QUB4156 and 3690:Mtub39 possessed eight and seven alleles, respectively. Furthermore, the four loci 2163b:QUB11b, 2996:MIRU26, 4052:QUB26, 580:MIRU04 ETRD, and 2401: Mtub30 separated the *M. bovis* isolates into six distinct alleles. The isolates were grouped into five distinct alleles by loci 960:MIRU10, 1644:MIRU16, and 2156: ETRA. Furthermore, the loci 424:Mtub04, 802:MIRU40, and 577:(ETRC) produced four unique alleles. In addition, the locus 1955:Mtub21 separated the isolates into three distinct alleles. It was evident that the locus 3192:M31 exhibited no heterogeneity and produced only one allele. According to diversity analysis, the discriminatory strength of the loci varied substantially, with (DI) ranging from 0.000 to 0.759.

**Table 6:** Results of tandem repeats with the implemented MIRU–VNTR loci, the specific allelic diversity, and the discriminatory index.

Locus	No. of strains per each (h) calculated with the various numbers of copies																			Allelic diversity (h)	Hunter–Gaston discriminatory index (DI)
	0	1	2	3	4	5	6	7	8	9	10	11	12	13	14	15	16	17	20		
424:Mtub04			30			5		3		2										0.65	0.4244
960:MIRU10		10	13	13		3						1								0.8	0.7385
1955:Mtub21		21	18			1														0.69	0.5346
2163b:QUB11b		1	1	9	19	9	1													0.81	0.6885
2996:MIRU26			1	8	5	22	1								3					0.78	0.6513
192:MIRU31 ETRE				40																0.74	0.000
4052:QUB26	2	5	12	15	3	3														0.85	0.759
4156:QUB4156		3	24	2	2	5					1				1			2		0.79	0.6256
580:MIRU04 ETRD		5	4	5	22	3				1										0.71	0.6667
1644:MIRU16			14	22	2	1		1												0.45	0.5859
802:MIRU 40		10	25	3		2														0.79	0.5526
2156:ETRA				13		5	2		3	17										0.81	0.7077
577:ETRC				2	10	26	2													0.72	0.5231
2401:Mtub30		3	14	8		12							2			1				0.72	0.7577
3690:Mtub39		2	14	16		3		2					2			1				0.75	0.7218

The discrimination power of the 15 loci varied significantly, with DI ranging from 0.000 to 0.759. After performing 15 MIRU–VNTR analyses, 35 genotypes were obtained based on the distribution of the allelic profile. The allelic diversity (h) of the loci utilized varied between 0.65 and 0.85. The loci MIRU10, QUB11b, MIRU26, QUB26, QUB4156, MIRU04, ETRA, Mtub30, and Mtub39 were highly discriminatory with (h) > 0.6, whereas the loci Mtub21, MIRU16, MIRU40, and ETRC were moderately discriminatory with (DI) > 0.5, and the locus Mtub04 was the least discriminatory with (h)< 0.5. One locus, MIRU31 ETRE, displayed no allelic diversity.

### 3.6 Results of the discrimination power of MIRU–VNTR loci combinations

**Figure 2:**
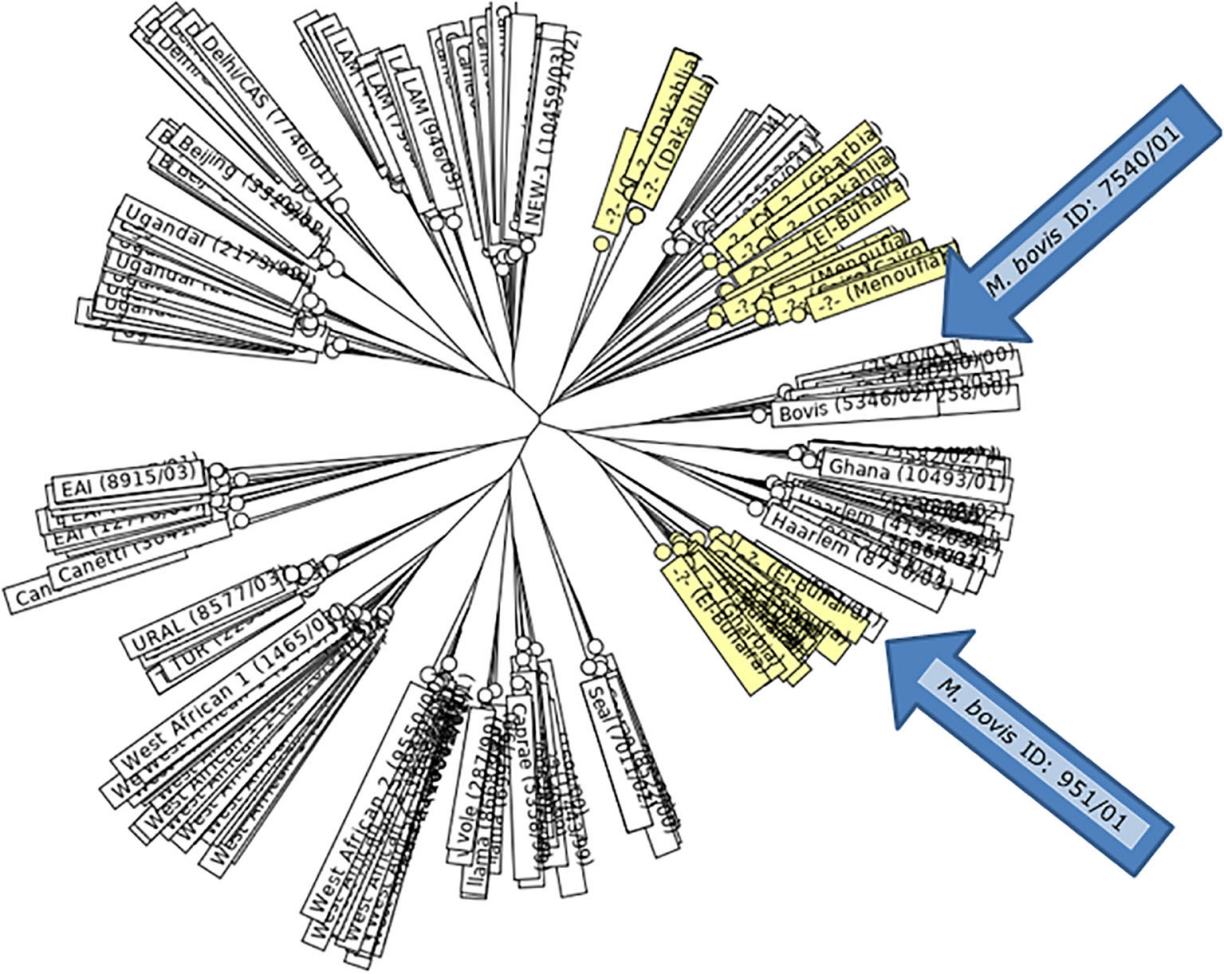
MIRU-VNTRplus: UPGMA-Tree-based Identification of the gained *M. bovis* isolates. The nearest international strain matches were *M. bovis* [(ID: 7540/01, Lineage: Bovis) and (ID: 951/01, Lineage: Bovis)] from Germany.

The 15 MIRU–VNTR loci resolved 40 *M. bovis* isolates to 35 different genotypes (DI = 0.9737), one of which was unique and 34 were clustered. The clusters contained one (n = 1) to two (n = 4) similar strains (**Table 7**, **Figure 2**). The total discrimination power of various loci combinations was confirmed. The nine highly discriminatory loci MIRU10, QUB11b, MIRU26, QUB26, QUB4156, MIRU04 ETRD, ETRA, Mtub30, and Mtub39 revealed that 35 genotypes were clustered into 23 clusters with a DI of 0.9676. The clusters contained one (n = 1) to two (n = 4) strains with similar characteristics. Furthermore, the second combination contained four moderately discriminatory loci, i.e., Mtub21, MIRU16, MIRU 40, and ETRC, which resolved the 40 isolates into 35 genotypes clustered into 15 clusters with a DI of 0.8894. These clusters contained one (n = 1) to two (n = 14) similar strains, and the combination of the Mtub04 and MIRU31 (ETRE) loci resolved the 40 isolates into 35 genotypes clustered into 4 clusters with a DI of 0.6111. These clusters contained one (n = 2) to two (n = 30) similar strains (**Table 5**). The nine highly discriminatory loci, i.e., MIRU10, QUB11b, MIRU26, QUB26, QUB4156, MIRU04 ETRD, ETRA, Mtub30, and Mtub39, are thought to be robust enough for exploratory molecular epidemiology research where quick findings are required. The employed loci combinations were effective predictors for isolate differentiation (95% CI 1.000–1.000).

**Table 7:** Assessment of the discriminating ability of various combinations of MIRU-VNTR loci.

Loci combinations		No. of gained clusters	No. of strains per single cluster	Hunter–Gaston discriminatory index (DI)	95% CI
1st	Mtub04, MIRU10, Mtub21, QUB11b, MIRU26, MIRU31ETRE, QUB26, QUB4156, MIRU04 ETRD, MIRU16, MIRU40, ETRA, ETRC, Mtub30, Mtub39	24	1–4	0.9737	1.000 (1.000–1.000)
2nd	MIRU10, QUB11b, MIRU26, QUB26, QUB4156, MIRU04 ETRD, ETRA, Mtub30, Mtub39	23	1–4	0.9676	1.000 (1.000–1.000)
3rd	Mtub21, MIRU16, MIRU40, ETRC	15	1–14	0.8894	1.000 (1.000–1.000)
4th	Mtub04, MIRU31 (ETRE)	4	1–30	0.6111	1.000 (1.000–1.000)

### 3.7 Association between isoniazid resistance and MIRU–VNTR analysis

Based on isoniazid resistance and MIRU–VNTR analysis, the ETRA locus expressed the highest number of copies in the genotypes coded 4 and 5 in cattle from Cairo, genotype coded 16 in cattle from Menoufia, and genotype coded 25 in cattle from Gharbia. However, the 3690:Mtub39 locus expressed the highest number of copies in the genotype coded 8 in cattle from Sharkia and genotype 34 in cattle from El-Buhaira. Moreover, the ETRC locus expressed the highest number of copies in the genotype coded 17 in cattle from Menoufia. Furthermore, the QUB4156 locus expressed the highest number of copies in the genotype coded 28 in cattle from Dakahlia. A statistically significant difference was found among the results of loci (p< 0.0001). Even though MIRU-VNTR analysis revealed no correlation between isoniazid resistance and genotypes, the ETRA, ETRC, and QUB4156 loci exhibited the highest number of copies among the genotypes of resistant isolates. Furthermore, there was no evidence of clonality in the distribution of isoniazid resistance among different genotypes.

### 3.8 Origin, existence, and rates of the gained MIRU–VNTR profiles

Regarding the obtained genotypes, the MIRU–VNTR analysis resolved 35 genotypes among the *M. bovis* isolates tested. Genotypes numbered 1 to 25 and 29 to 35 were evenly distributed among the isolates at a rate of 1/40 (2.5%). Furthermore, genotypes 26 and 27 expressed similar frequency among isolates from the cattle of Dakahlia district, with a frequency of 3/40 (7.5%) for each. Moreover, genotype 28 was prevalent among isolates from the cattle of Dakahlia district at a rate of 2/40 (5%), (**Table 8**). Based on the similarities in genotypes, 15 MIRU-VNTR loci, and the MIRU-VNTRplus database, *M. bovis*, ID: 7540/01, Lineage: Bovis and ID: 951/01, Lineage: Bovis from Germany were the closest lineages to the genotypes identified in the present study.

**Table 8:** Distribution of allelic profiles for isolates after the investigation of the MIRU-VNTR loci.

Isolate ID	Origin	Species	Used 15 MIRU-VNTR loci														
			424	960	1955	2163b	2996	3192	4052	4156	580	1644	802	2165	577	2401	3690
1	*Cairo*	*Cattle*	2	3	2	4	5	3	5	2	4	3	2	6	5	5	2
2	*Cairo*	*Cattle*	5	3	2	5	4	3	2	2	2	3	2	3	5	2	2
3	*Cairo*	*Cattle*	2	3	1	6	3	3	2	2	2	2	2	5	4	5	2
4	*Cairo*	*Cattle*	5	2	1	3	6	3	4	5	4	2	2	8	5	5	2
5	*Cairo*	*Cattle*	2	2	1	3	4	3	4	2	4	2	2	8	5	1	2
6	*Sharkia*	*Buffalo*	2	3	1	3	5	3	3	2	2	3	2	9	5	3	2
7	*Sharkia*	*Cattle*	2	2	2	4	5	3	2	2	4	3	1	9	5	5	3
8	*Sharkia*	*Cattle*	2	3	1	5	5	3	3	14	4	2	2	5	5	2	12
9	*Menoufia*	*Cattle*	2	3	1	4	2	3	0	2	1	3	3	3	3	3	2
10	*Menoufia*	*Cattle*	2	3	5	3	3	3	4	3	9	7	2	3	5	12	7
11	*Menoufia*	*Buffalo*	2	3	1	3	3	3	5	3	3	2	1	8	4	15	7
12	*Menoufia*	*Cattle*	2	3	1	4	5	3	0	10	3	2	5	3	4	1	3
13	*Menoufia*	*Cattle*	2	2	2	4	5	3	3	2	4	3	2	9	5	5	3
14	*Menoufia*	*Cattle*	2	3	1	3	5	3	3	2	4	4	2	5	6	2	5
15	*Menoufia*	*Buffalo*	2	2	2	4	5	3	1	4	4	3	2	3	5	5	2
16	*Menoufia*	*Cattle*	2	2	2	4	5	3	3	2	4	3	2	9	5	5	3
17	*Menoufia*	*Cattle*	2	3	1	3	5	3	3	2	4	4	2	5	6	2	5
18	*Gharbia*	*Cattle*	2	2	2	4	5	3	3	2	4	3	2	9	5	5	3
19	*Gharbia*	*Cattle*	2	1	1	4	5	3	2	2	1	3	2	9	5	12	3
20	*Gharbia*	*Cattle*	2	1	2	4	5	3	1	2	4	2	2	9	5	3	3
21	*Gharbia*	*Cattle*	2	1	1	1	3	3	2	5	3	5	5	3	4	1	12
22	*Gharbia*	*Cattle*	2	11	2	4	5	3	3	2	4	3	2	5	5	5	3
23	*Gharbia*	*Cattle*	2	2	2	4	5	3	3	2	4	3	2	9	5	5	3
24	*Gharbia*	*Cattle*	2	1	2	4	5	3	1	2	4	3	2	9	5	5	3
25	*Gharbia*	*Cattle*	2	9	2	4	5	3	1	2	4	3	2	9	5	5	3
26	*Dakahlia*	*Cattle*	5	5	1	5	14	3	2	5	5	2	1	3	5	2	2
27	*Dakahlia*	*Cattle*	5	5	1	5	14	3	2	5	5	2	1	3	5	2	2
28	*Dakahlia*	*Cattle*	5	5	1	5	14	3	2	5	5	2	1	3	5	2	2
29	*Dakahlia*	*Cattle*	7	2	1	5	3	3	2	1	4	3	1	9	4	2	2
30	*Dakahlia*	*Cattle*	7	2	1	5	3	3	2	1	4	3	1	9	4	2	2
31	*Dakahlia*	*Cattle*	7	2	1	5	3	3	2	1	4	3	1	9	4	2	2
32	*Dakahlia*	*Cattle*	9	1	1	3	4	3	3	17	1	2	3	3	4	3	1
33	*Dakahlia*	*Cattle*	9	1	1	3	4	3	3	17	1	2	3	3	4	3	1
34	*El-Buhaira*	*Cattle*	2	3	2	5	4	3	5	2	3	2	1	3	4	3	5
35	*El-Buhaira*	*Cattle*	2	2	2	4	5	3	3	2	4	3	2	9	5	2	3
36	*El-Buhaira*	*Cattle*	2	1	2	4	5	3	3	2	4	3	2	9	5	2	3
37	*El-Buhaira*	*Cattle*	2	1	2	4	5	3	1	2	4	3	2	9	5	2	3
38	*El-Buhaira*	*Buffalo*	2	2	2	2	5	3	3	2	1	3	2	9	5	2	3
39	*El-Buhaira*	*Cattle*	2	1	2	4	3	3	3	2	2	2	1	6	5	3	15
40	*El-Buhaira*	*Cattle*	2	1	1	4	5	3	2	4	3	3	2	3	3	3	3

## 4 Discussion

Bovine tuberculosis caused by *M. bovis* is considered a zoonotic organism with cattle being the primary cause of infection in humans (WOAH, 2024). *M. bovis* is responsible for 10% to 20% of human tuberculosis cases in Africa and other underdeveloped countries (Wernery and Kinne, 2012). As *M. bovis* is typically acquired through oral ingestion and gastrointestinal sickness is a common clinical sign, the global prevalence of *M. bovis* infection is likely higher due to the development of extrapulmonary tuberculosis (Elsohaby et al., 2020). As a result of eradication programs and pasteurization of milk, the number of *M. bovis*-associated human TB cases has decreased significantly in the developed countries. However, *M. bovis* remains a zoonotic threat to humans who come into contact with sick animals (Cosivi et al., 1998).

Tuberculin test result of this study was 2.7%, lower than the recent report from Egypt, which indicated a 3.4% positivity rate using the single intradermal comparative skin tuberculin test for dairy cattle (Abdelaal et al., 2019). This decrease was attributed to the stringent application of the General Authority of Veterinary Services’ test and slaughter policy and the Egyptian Ministry of Agriculture goals to control animal infection with *M. bovis* and its potential transmission to humans (Gedawy et al., 2021). Another reason for this difference could be attributed to errors in tuberculin testing procedures during implementation of the national control program which may have resulted in missing infected cases (Elsayed et al., 2016).

Upon postmortem examination, animals from Cairo, Sharkia, Menoufia, Dakahlia, and El-Buhaira districts expressed generalized lesions, while those from Gharbia primarily had pulmonary lesions. This difference in lesion distribution is likely due to the route of *M. bovis* infection, a finding consistent with previous research (Serrano et al., 2018) indicating that respiratory infections cause respiratory lesions and oral infections lead to digestive tract lesions.

Compared to buffalo, cattle in this study had more positive tuberculin tests and *M. bovis* isolation, aligning with findings by Elsayed et al. (2022). This is likely due to Egypt importing many cattle, some of which might have been early *M. bovis* carriers (3–6 weeks, a pre-allergic stage leading to false negatives with tuberculin test) or were experiencing anergy from severe infection, also causing false negatives. Other factors contributing to false negative results include recent PPD administration (8–60 days prior), old age, early postpartum cows, low-potency tuberculin, subcutaneous rather than intradermal injection, or contaminated tuberculin (Borham et al., 2022a).

All obtained isolates (40 out of 40 [100%]) were confirmed to be members of the MTBC using *IS*6110 primer in PCR test, and *M. bovis* was detected using primers targeting genomic RDs (RD1, 4, 9, and 12), as *M. bovis* is characterized by the presence of RD1 at 146 bp. These results demonstrate that genotypic analysis of different RD regions was essential for identifying the genetic variation of closely related MTBC members (Thacker et al., 2011).

The Egyptian national authorities focus on detection of *M. bovis* from farm animals (Lasserre et al., 2015). Some efforts are also directed toward the detection of other MTBC bacteria on Egyptian farms. Research efforts are also directed toward the detection of other MTBC members such as in 2016, the identification of *M. bovis* and *M. kansasii* in farm animals and the presence of isoniazid and rifampicin-resistant *M. tuberculosis* isolates from cattle and buffaloes during the years 2016 and 2022 (Abdel-Moein et al., 2016; Olea-Popelka et al., 2017; Borham et al., 2022b).

We didn’t use samples from all tuberculin-positive animals in the further steps of this study because they didn’t yield *M. bovis* isolates. Of the 65 tuberculin-positive cases, only 40 (61.53%) were positive for *M. bovis* isolation and were confirmed by PCR. We specifically needed these confirmed isolates for further tests like phenotypic and genotypic antimicrobial susceptibility testing, detecting mutations in resistance genes, and MIRU-VNTR typing. In addition to that, tissue samples from isolation-negative animals, when homogenized, can limit the amount of mycobacterial DNA recovered and may inhibit PCR, as confirmed by Taylor et al. (2007).

Animal *Mycobacterium* isolates and *M. bovis* are rarely tested for drug susceptibility. However, bTB continues to be a human health hazard, especially in low-income and bTB-endemic areas (WHO, 2011; Wernery and Kinne, 2012).

The World Health Organization has recently approved colorimetric tests for testing the antimicrobial susceptibility of *Mycobacterium tuberculosis* complex members, which are specific, highly sensitive, quick, and cost-effective procedures that use specific reagents to cause a color change (Campanerut et al., 2011). The indirect resazurin microtiter assay, which involves reducing the colored dye resazurin was effectively tested on human isolates of *M. tuberculosis* cultivated in a microtiter plate with liquid culture medium containing antituberculosis medications (Telenti et al., 1997). All obtained isolates (40 out of 40 [100%]) were susceptible to rifampicin, ethambutol, streptomycin, ciprofloxacin, levofloxacin, ofloxacin, and sparfloxacin. While 80% (32/40) of the isolates were susceptible to isoniazid, a notable 20% (8/40) of them demonstrated resistance. This resistance to either rifampicin, isoniazid, or both has already been identified in *M. bovis* isolates from cattle (Sechi et al., 2001; Djemal et al., 2018).

DNA from the eight isoniazid-resistant isolates was exposed to PCR using specific primers to amplify *kat*G to find out which mutations were involved in the *M. bovis* resistance to isoniazid. Isolates resistant to isoniazid showed three different *kat*G mutations; both cattle isolates from Cairo exhibited mutations in codon 315 with an AGC→ACC alteration leading to the Ser→Thr mutation, which is important for *M. bovis* isoniazid resistance (Shim et al., 1997; Sechi et al., 2001; Djemal et al., 2018). Furthermore, one cattle isolate from Sharkia and two cattle isolates from Menoufia showed mutations in codon 463. They showed CTG→CGG alteration, which is normally present in the *M. tuberculosis* genome and some *M. bovis* and *M. microti* strains, suggesting its existence in wild-type strains, and the presence of this mutation in our isolates may be a polymorphism not related to isoniazid resistance (Yang et al., 2015). Moreover, three cattle isolates from Gharbia, Dakahlia, and El-Buhaira had the same mutation in codon 506 with a GAG→AAG change.

The involvement of these mutations in determining isoniazid resistance in *M. bovis* should be investigated in the *M. bovis* isolates from the Delta area. Despite limited local studies on *kat*G mutations linked to isoniazid resistance in *M. bovis* within Egypt’s Delta area (especially compared to *M. tuberculosis*), a recent report from Egypt (Soliman et al., 2024) revealed a significant finding: a *kat*G codon 463 point mutation was present in 100% (5 out of 5) of *M. bovis* isolates from human patients in the Delta, indicating this mutation may be responsible for their isoniazid resistance. This finding suggests that mutations may be widespread, yet limited research has prevented us from observing their true frequency.

We investigated the allelic diversity (h) and DI of 15 MIRU-VNTR markers. Our results, with ‘h’ values between 0.65 and 0.85, closely mirrored those of Elsayed (2019), who also used the same set of markers and reported an ‘h’ range of 0.64 to 0.86. Notably, for Mtub04, Mtub30, and Mtub39, our ‘h’ indices exceeded those found in a Chinese study (Belakehal et al., 2022). Furthermore, MIRU10, QUB11b, MIRU26, MIRU31, QUB26, MIRU04, MIRU16, MIRU 40, ETRA, and ETRC all showed higher ‘h’ values compared to both Chinese and Algerian studies (Belakehal et al., 2022). A significant number of markers (MIRU10, QUB11b, MIRU26, QUB26, QUB4156, MIRU04, ETRA, Mtub30, and Mtub39) proved to be highly discriminatory (DI > 0.6), consistent with a recent Egyptian study (Elsayed, 2019). In contrast, we found that Mtub21, MIRU16, MIRU 40, and ETRC were only moderately discriminatory (DI > 0.5), which differed from Elsayed (2019) report of high discrimination (DI > 0.6). Similarly, Mtub04 showed the lowest discriminatory power (DI< 0.5) in our study, contradicting the higher discriminatory power (DI > 0.6) reported by Elsayed (2019).

In this research, our initial combination of 15 MIRU-VNTR loci achieved a strong DI of 0.9737. This result is notably higher than the DI of 0.9641 reported in a recent Egyptian study (Elsayed, 2019) and significantly better than the 0.897 DI from a Chinese study (Belakehal et al., 2022). Our results were, however, similar to a recent Algerian study’s DI of 0.9779 (Belakehal et al., 2022). Furthermore, our second combination of 10 loci showed a higher DI (0.9676) than a comparable six loci combination in the Egyptian study. While our third combination (four loci) had a lower DI than the Egyptian study’s seven loci combination, our fourth combination (two loci) still produced a superior DI compared to Elsayed (2019) findings. This strongly suggests that employing the full 15 MIRU-VNTR loci significantly improves genotyping resolution over the previously common 12-locus approach.

Modern combinations of these loci have significantly boosted their ability to distinguish between strains. A DI of 0.95 is considered the ideal threshold for reliable molecular epidemiological studies, and the latest MIRU-VNTR loci selection in our study surpasses this, achieving a DI of 0.9737. This suggests that a specific set of 9 loci (MIRU10, QUB11b, MIRU26, QUB26, QUB4156, MIRU04 ETRD, ETRA, Mtub30, and Mtub39) could serve as a valuable first-line tool for quick data collection in molecular epidemiology as its DI was 0.9676; potentially predicting comprehensive genotyping results from the full MIRU-VNTR panel for more detailed investigations.

Our analysis of 40 isolates using 15 MIRU–VNTR loci, all showing some genetic variation, revealed 35 unique genotypes with a high DI of 0.9737. Among these, one genotype was distinct, and the remaining 34 formed clusters, each containing one to four similar strains. Most genotypes (G1–25 and G29-35) were found in a relatively low frequency, appearing in 1 out of 40 isolates (2.5%) each. However, genotypes G26 and G27 were more common, each representing 3 out of 40 isolates (7.5%) from cattle in Dakahlia district. Genotype G28 was also notable in the same area, occurring in 2 out of 40 isolates (5%). When we compared our genotypes to the MIRU–VNTRplus database, the closest matches were *M. bovis* (ID: 7540/01, Lineage: Bovis) and (ID: 951/01, Lineage: Bovis) from Germany. These findings call for intensive and strict testing of cattle imported from Germany for *M. bovis* infection.

It would be interesting for further research to see if some of these genotypes are identified in human cases in the future and if so in which geographical areas.

Interestingly, while we did not find a direct link between isoniazid resistance and specific genotypes, the ETRA, ETRC, and QUB4156 loci showed the highest number of copies in genotypes from resistant isolates. Furthermore, the distribution of isoniazid resistance among various genotypes did not express clonality.

## 5 Conclusions

*M. bovis* isolates from animal origin should be subjected to antitubercular drug susceptibility testing to assess the probability of drug-resistant genotypes in the Delta area that could be transferred to humans. Some *M. bovis* isolates demonstrated resistance to isoniazid, whereas all isolates were susceptible to rifampicin, ethambutol, and streptomycin. The isolates expressed susceptibility to the following quinolone antimicrobials: ciprofloxacin, levofloxacin, ofloxacin, and sparfloxacin. The detection and sequencing of resistance genes will provide evidence regarding the mutation responsible for such isoniazid resistance, the finding of the *kat*G mutations in codon 315 is considered significant for this resistance. The MIRU10, QUB11b, MIRU26, QUB26, QUB4156, MIRU04 ETRD, ETRA, Mtub30, and Mtub39 loci combination may be useful for acquiring rapid data and understanding first-line molecular epidemiology. This research has improved our understanding of *M. bovis*’s diversity and revealed that antimicrobial resistance may be caused by accumulating mutations. In addition, this research provides essential tools for a rapid and accurate diagnosis of *M. bovis* among animals and humans. We believe that these findings will support the development of an effective treatment program for humans in the case of *M. bovis* zoonotic infection in the Delta area.

## Data Availability

The datasets presented in this study can be found in online repositories. The names of the repository/repositories and accession number(s) can be found in the article/**supplementary material**.
