# Supplementary material for: Genetic diversity and antibiotic resistance of Mycobacterium bovis in bovines in the Delta area of Egypt
Source: Front Cell Infect Microbiol. 2025 Sep 12;15:1600225. doi: 10.3389/fcimb.2025.1600225 (PMC12463954; doi:10.3389/fcimb.2025.1600225)
Supplement: Supplementary file 1 [file Table1.docx]

Supplementary Material

**SUPPLEMENTARY TABLE 1 Phenotypic antimicrobial susceptibility testing using broth dilution method.**

| **Locality** | **Isolates** | **Animal species** | | **Isolate code**  **and origin** | **Antimicrobial susceptibility testing** | | | | |
| --- | --- | --- | --- | --- | --- | --- | --- | --- | --- |
|  |  |  |  |  | **Isoniazid (INH, 1 μg/mL)** | **Rifampicin (RIF, 1 μg/mL)** | **Ethambutol (EMB, 5 μg/mL)** | | **Streptomycin (STR, 2 μg/mL)** |
| Cairo | 5 | Cattle | | 1 | S | S | S | | S |
|  |  | Cattle | | 2 | S | S | S | | S |
|  |  | Cattle | | 3 | S | S | S | | S |
|  |  | Cattle | | 4 | R | S | S | | S |
|  |  | Cattle | | 5 | R | S | S | | S |
| Sharkia | 3 | Buffalo | | 6 | S | S | S | | S |
|  |  | Cattle | | 7 | S | S | S | | S |
|  |  | Cattle | | 8 | R | S | S | | S |
| Menoufia | 9 | Cattle | | 9 | S | S | S | | S |
|  |  | Cattle | | 10 | S | S | S | | S |
|  |  | Buffalo | | 11 | S | S | S | | S |
|  |  | Cattle | | 12 | S | S | S | | S |
|  |  | Cattle | | 13 | S | S | S | | S |
|  |  | Cattle | | 14 | S | S | S | | S |
|  |  | Buffalo | | 15 | S | S | S | | S |
|  |  | Cattle | | 16 | R | S | S | | S |
|  |  | Cattle | | 17 | R | S | S | | S |
| Gharbia | 8 | Cattle | | 18 | S | S | S | | S |
|  |  | Cattle | | 19 | S | S | S | | S |
|  |  | Cattle | | 20 | S | S | S | | S |
|  |  | Cattle | | 21 | S | S | S | | S |
|  |  | Cattle | | 22 | S | S | S | | S |
|  |  | Cattle | | 23 | S | S | S | | S |
|  |  | Cattle | | 24 | S | S | S | | S |
|  |  | Cattle | | 25 | R | S | S | | S |
| Dakahlia | 8 | Cattle | | 26 | S | S | S | | S |
|  |  | Cattle | | 27 | S | S | S | | S |
|  |  | Cattle | | 28 | S | S | S | | S |
|  |  | Cattle | | 29 | R | S | S | | S |
|  |  | Cattle | | 30 | S | S | S | | S |
|  |  | Cattle | | 31 | S | S | S | | S |
|  |  | Cattle | | 32 | S | S | S | | S |
|  |  | Cattle | | 33 | S | S | S | | S |
| El-Buhaira | 7 | Cattle | | 34 | R | S | S | | S |
|  |  | Cattle | | 35 | S | S | S | | S |
|  |  | Cattle | | 36 | S | S | S | | S |
|  |  | Cattle | | 37 | S | S | S | | S |
|  |  | Buffalo | | 38 | S | S | S | | S |
|  |  | Cattle | | 39 | S | S | S | | S |
|  |  | Cattle | | 40 | S | S | S | | S |
| Sensitive (S) |  | |  | | 32/40  (80%) | 40/40  (100%) | 40/40  (100%) | 40/40  (100%) | |
| Resistant (R) |  | |  | | 8/40  (20 % ) | 0/40  (0.0%) | 0/40  (0.0%) | 0/40  (0.0%) | |

**SUPPLEMENTARY TABLE 2 Antimicrobial susceptibility testing against quinolones.**

| **Locality** | **Isolates** | **Animal species** | **Isolate code**  **and origin** | **Antimicrobial susceptibility testing** | | | |
| --- | --- | --- | --- | --- | --- | --- | --- |
|  |  |  |  | **Ciprofloxacin (CPFX , 1 μg/mL)** | **Levofloxacin (LVX, 0.5 μg/mL)** | **Ofloxacin**  **(OFX,**  **1 μg/mL)** | **Sparfloxacin**  **(SPFX, 1 μg/mL)** |
| Cairo | 5 | Cattle | 1 | S | S | S | S |
|  |  | Cattle | 2 | S | S | S | S |
|  |  | Cattle | 3 | S | S | S | S |
|  |  | Cattle | 4 | S | S | S | S |
|  |  | Cattle | 5 | S | S | S | S |
| Sharkia | 3 | Buffalo | 6 | S | S | S | S |
|  |  | Cattle | 7 | S | S | S | S |
|  |  | Cattle | 8 | S | S | S | S |
| Menoufia | 9 | Cattle | 9 | S | S | S | S |
|  |  | Cattle | 10 | S | S | S | S |
|  |  | Buffalo | 11 | S | S | S | S |
|  |  | Cattle | 12 | S | S | S | S |
|  |  | Cattle | 13 | S | S | S | S |
|  |  | Cattle | 14 | S | S | S | S |
|  |  | Buffalo | 15 | S | S | S | S |
|  |  | Cattle | 16 | S | S | S | S |
|  |  | Cattle | 17 | S | S | S | S |
| Gharbia | 8 | Cattle | 18 | S | S | S | S |
|  |  | Cattle | 19 | S | S | S | S |
|  |  | Cattle | 20 | S | S | S | S |
|  |  | Cattle | 21 | S | S | S | S |
|  |  | Cattle | 22 | S | S | S | S |
|  |  | Cattle | 23 | S | S | S | S |
|  |  | Cattle | 24 | S | S | S | S |
|  |  | Cattle | 25 | S | S | S | S |
| Dakahlia | 8 | Cattle | 26 | S | S | S | S |
|  |  | Cattle | 27 | S | S | S  S | S |
|  |  | Cattle | 28 | S | S |  | S |
|  |  | Cattle | 29 | S | S | S | S |
|  |  | Cattle | 30 | S | S | S | S |
|  |  | Cattle | 31 | S | S | S | S |
|  |  | Cattle | 32 | S | S | S | S |
|  |  | Cattle | 33 | S | S | S | S |
| El-Buhaira | 7 | Cattle | 34 | S | S | S | S |
|  |  | Cattle | 35 | S | S | S | S |
|  |  | Cattle | 36 | S | S | S | S |
|  |  | Cattle | 37 | S | S | S | S |
|  |  | Buffalo | 38 | S | S | S | S |
|  |  | Cattle | 39 | S | S | S | S |
|  |  | Cattle | 40 | S | S | S | S |
| Sensitive (S) |  |  | | 40/40  (100%) | 40/40  (100%) | 40/40  (100%) | 40/40  (100%) |
| Resistant (R) |  |  | | 0/40  (0.0%) | 0/40  (0.0%) | 0/40  (0.0%) | 0/40  (0.0%) |

**SUPPLEMENTARY TABLE 3 Primers of the antimicrobial resistance intended for sequencing applied in this study.**

| **Antimicrobial** | **Specific gene** | **Annealing temperature C°**  **and (cycles)** | **Amplicon**  **size** | **Sequence** |
| --- | --- | --- | --- | --- |
| Rifampicin | *rpo*B | 55 (40) | 157 | F:TGCACGTCGCGGACCTCCA  R:TCGCCGCGATCAAGGAGT |
| Isoniazid | *inh*A | 60 (40) | 427 | F:ACAAGGACGCACATGACAG  R:GCGCTGCTGCCGATCATGA |
|  | *kat*G | 55 (35) | 337 | F:GTGCCCGAGCAACACCCACC  R:TAGGTGCCGGCAGCGTGCCA |
|  | *ahp*C | 50 (35) | 455 | F:CGGTCCTCGAACTCGTC  R:GCAACGTCGACTGGCTC |
| Streptomycin | *rps*L | 61 (35) | 306 | F:CCCACCATTCAGCAGCTGGT  R:GTCGAGCGAACCGCGAATGA |
|  | *rrs* | 61 (35) | 238 | F:GTAGTCCACGCCGTAAACGG  R:AGGCCACAAGGGAACGCCTA |
| Ethambutol | *emb*B | 55 (35) | 399 | F:ACAGACTGGCGTCGCTGACA  R:ACGCTGAAACTGCTGGCGAT |
